# l-Serine methyl ester hydro­chloride

**DOI:** 10.1107/S1600536809046480

**Published:** 2009-11-07

**Authors:** Arie Schouten, Martin Lutz

**Affiliations:** a Bijvoet Center for Biomolecular Research, Crystal and Structural Chemistry, Faculty of Science, Utrecht University, Padualaan 8, 3584 CH Utrecht, The Netherlands

## Abstract

In the enanti­opure crystal of the title compound, C_4_H_10_NO_3_^+^·Cl^−^, inter­molecular O—H⋯Cl and N—H⋯Cl hydrogen bonds link the mol­ecules into layers parallel to (001).

## Related literature

Esterification of the carboxyl group of amino acids plays an important role in the synthesis of peptides, especially due to the increased solubility in non-aquous organic solvents, see: Bodanszky (1993 ▶). For related structures, see: Bryndal *et al.* (2006 ▶); Görbitz (1989 ▶). For the determination of the absolute structure, see: Flack & Bernardinelli (2000 ▶); Flack & Shmueli (2007 ▶); Hooft *et al.* (2008 ▶).
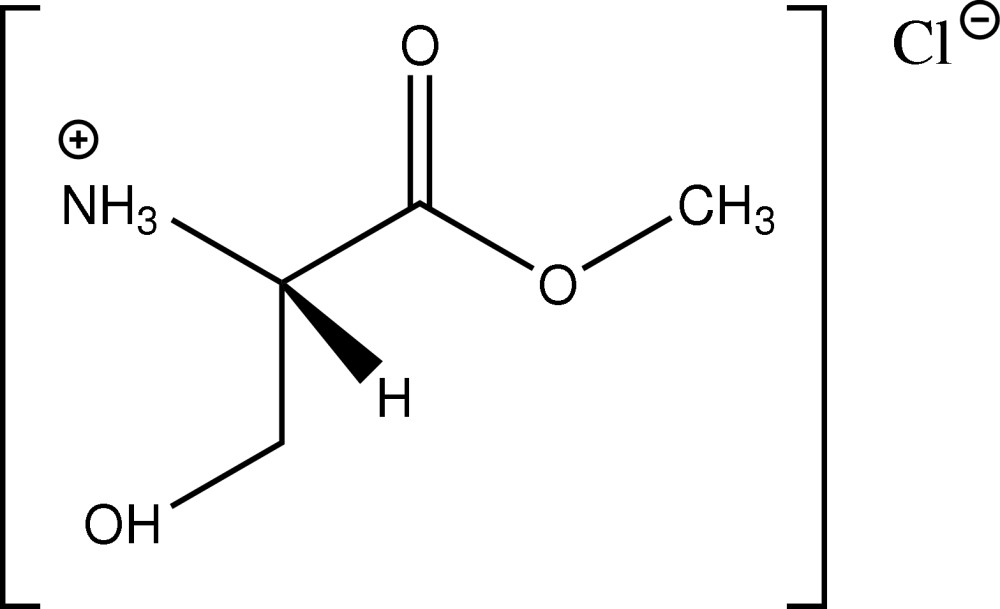


## Experimental

### 

#### Crystal data

C_4_H_10_NO_3_^+^·Cl^−^
*M* *_r_* = 155.58
Monoclinic, 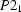

*a* = 5.22645 (9) Å
*b* = 6.39388 (14) Å
*c* = 11.6420 (4) Å
β = 90.090 (1)°
*V* = 389.04 (2) Å^3^
*Z* = 2
Mo *K*α radiation
μ = 0.44 mm^−1^
*T* = 150 K
0.38 × 0.33 × 0.15 mm

#### Data collection

Nonius KappaCCD diffractometer
Absorption correction: multi-scan (*SADABS*; Sheldrick, 2002 ▶) *T*_min_ = 0.78, *T*_max_ = 0.93
15456 measured reflections
3449 independent reflections
3242 reflections with *I* > 2σ(*I*)
*R*_int_ = 0.030

#### Refinement

*R*[*F* ^2^ > 2σ(*F* ^2^)] = 0.022
*wR*(*F* ^2^) = 0.058
*S* = 1.07
3449 reflections
100 parameters
1 restraint
H atoms treated by a mixture of independent and constrained refinement
Δρ_max_ = 0.25 e Å^−3^
Δρ_min_ = −0.17 e Å^−3^
Absolute structure: Flack (1983 ▶), 1599 Friedel pairs
Flack parameter: 0.00 (3)

### 

Data collection: *COLLECT* (Nonius, 1999 ▶); cell refinement: *PEAKREF* (Schreurs, 2005 ▶); data reduction: *EVAL15* (Xian *et al.*, 2006 ▶) and *SADABS* (Sheldrick, 2002 ▶); program(s) used to solve structure: *SHELXS97* (Sheldrick, 2008 ▶); program(s) used to refine structure: *SHELXL97* (Sheldrick, 2008 ▶); molecular graphics: *PLATON* (Spek, 2009 ▶); software used to prepare material for publication: *PLATON*.

## Supplementary Material

Crystal structure: contains datablocks I, global. DOI: 10.1107/S1600536809046480/vm2012sup1.cif

Structure factors: contains datablocks I. DOI: 10.1107/S1600536809046480/vm2012Isup2.hkl

Additional supplementary materials:  crystallographic information; 3D view; checkCIF report

## Figures and Tables

**Table 1:** Hydrogen-bond geometry (Å, °)

*D*—H⋯*A*	*D*—H	H⋯*A*	*D*⋯*A*	*D*—H⋯*A*
N1—H1*A*⋯Cl1	0.867 (16)	2.390 (16)	3.2237 (8)	161.2 (14)
N1—H1*B*⋯Cl1^i^	0.825 (18)	2.336 (18)	3.1563 (8)	172.7 (16)
N1—H1*C*⋯Cl1^ii^	0.865 (14)	2.264 (14)	3.0979 (7)	161.9 (12)
O3—H3⋯Cl1^iii^	0.847 (18)	2.261 (18)	3.1041 (7)	173.9 (15)

Symmetry codes: (i) 
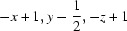
; (ii) 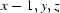; (iii) 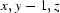.

**Fig. 1.**
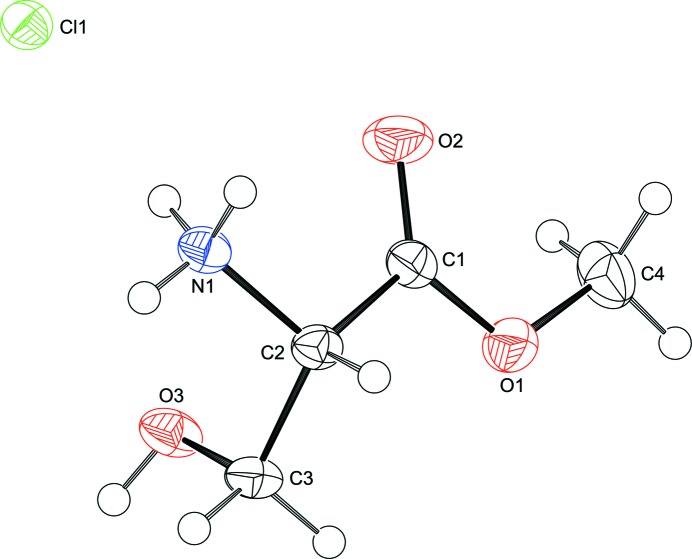
Molecular structure of (I), with atom labels and 50% probability displacement ellipsoids for non-H atoms.

**Fig. 2.**
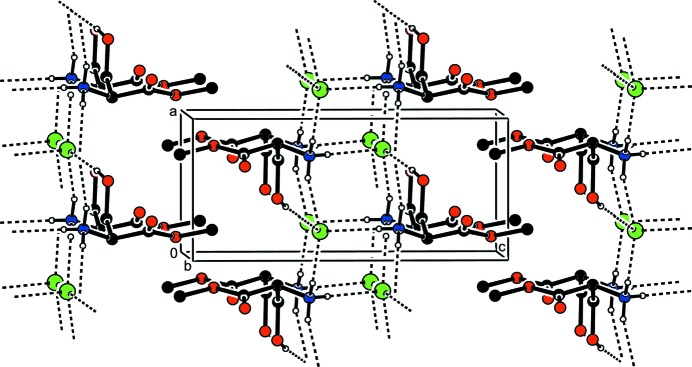
The packing of (I), viewed down the b axis, showing sheets running parallel to (001) with molecules connected by O—H···Cl and N—H···Cl hydrogen bonds (dashed lines). H atoms not involved in hydrogen bonding have been omitted for clarity.

## supplementary crystallographic information

### Comment

Esterification of the carboxyl group of amino acids plays an important role in the synthesis of peptides, especially due to the increased solubility in non-aquous organic solvents (Bodanszky, 1993). The synthesis of methyl esters is straightforward and can be performed by the reaction of HCl gas with a suspension of the amino acid in methanol. In this reaction the hydrochloride of the amino acid methyl ester is obtained, which is the subject of the present study.

A molecular plot of the title compound (I) is shown in Fig. 1. As a consequence of the protection of the carboxyl group, only the amino group is available as protonation site. The O1—C1—C2—N1 and C4—O1—C1—C2 torsion angles of -175.99 (7) and 179.72 (7) ° indicate an extended structure of the backbone. Nevertheless, we do not see a stabilization of this extended structure by an intramolecular hydrogen bond between the ammonium group and O2. Such a stabilization would require a N—H bond in the plane of O2—C1—C2—N1, which is not the case here. Similar extended structures are also found in the methyl ester hydrochlorides of L-cysteine (Görbitz, 1989) and L-tyrosine (Bryndal *et al.*, 2006). Bond lengths and angles in (I) are as expected (Table 1).

With three H-atoms at N1 and one H-atom at O3 the molecule has four hydrogen bond donors. Three oxygen atoms and the chloride anion could act as hydrogen bond acceptors. In fact, only the chloride is used as a hydrogen bond acceptor, here (Table 2). This results in an infinite two-dimensional hydrogen bonding network parallel to the *ab* plane, as shown in Fig. 2. In the *c* direction the hydrogen-bonded planes are separated by hydrophobic OCH_3_ groups. Interestingly, the two-dimensional motif is also reflected in the morphology of the crystal, where (001) has the smallest dimension.

Despite a β-angle of 90.090 (1)° there is no orthorhombic symmetry in this crystal structure. The *R*_int_ value for Laue-symmetry *mmm* is 31% compared to 2% for 2/*m*. We also did not find indications for pseudo-orthorhombic twinning. The reflections in the diffraction images were not split, and an analysis of the F_o_/F_c_ listing with the TWINROTMAT routine of *PLATON* (Spek, 2009) did not suggest the presence of twinning.

Because (I) is derived from enantiopure L-serine, the absolute configuration was known in advance. Nevertheless, the presence of chloride provides enough enantiomorph distinguishing power (Friedif = 123, Flack & Shmueli, 2007) to allow a reliable experimental confirmation of the absolute structure. This was done using the Flack parameter (Flack, 1983), which resulted in *x* = 0.00 (3), and the Hooft parameter (Hooft *et al.*, 2008), which resulted in *y* = 0.005 (15). As expected, the standard uncertainty of the Hooft parameter is significantly lower than in the Flack parameter, but both parameters confirm the correct absolute structure of (I).

### Experimental

0.4 g of L-serine methyl ester hydrochloride (obtained commercially from Aldrich) was dissolved in 10 ml absolute ethanol followed by slow evaporation at room temperature. Single crystals suitable for X-ray diffraction were obtained after a final seeding step by adding a tiny amount of solid starting material.

### Refinement

All H atoms were located in difference Fourier maps. H atoms bonded to N and O atoms were refined freely with isotropic displacement parameters. H(C) atoms were refined using a riding model (including free rotation of the methyl substituents), with C—H = 0.95–1.00 Å and with *U*_iso_(H) = 1.2 (1.5 for methyl groups) times *U*_eq_(C).

Friedel pairs were kept separate during the refinement and the Flack parameter was included in the least-squares matrix using TWIN/BASF instructions in *SHELXL97*. This has been shown to reduce the uncertainty of the Flack parameter compared to the hole-in-one algorithm (Flack & Bernardinelli, 2000).

### Crystal data

**Table:**

C_4_H_10_NO_3_^+^·Cl^−^	*F*(000) = 164
*M**_r_* = 155.58	*D*_x_ = 1.328 Mg m^−^^3^
Monoclinic, *P*2_1_	Mo *K*α radiation, λ = 0.71073 Å
Hall symbol: P 2yb	Cell parameters from 14244 reflections
*a* = 5.22645 (9) Å	θ = 1.8–35.0°
*b* = 6.39388 (14) Å	µ = 0.44 mm^−^^1^
*c* = 11.6420 (4) Å	*T* = 150 K
β = 90.090 (1)°	Irregular plate, colourless
*V* = 389.04 (2) Å^3^	0.38 × 0.33 × 0.15 mm
*Z* = 2	

### Data collection

**Table:**

Nonius KappaCCD diffractometer	3449 independent reflections
Radiation source: rotating anode	3242 reflections with *I* > 2σ(*I*)
graphite	*R*_int_ = 0.030
φ and ω scans	θ_max_ = 35.0°, θ_min_ = 1.8°
Absorption correction: multi-scan (*SADABS*; Sheldrick, 2002)	*h* = −8→8
*T*_min_ = 0.78, *T*_max_ = 0.93	*k* = −10→10
15456 measured reflections	*l* = −18→18

### Refinement

**Table:**

Refinement on *F*^2^	Secondary atom site location: difference Fourier map
Least-squares matrix: full	Hydrogen site location: difference Fourier map
*R*[*F*^2^ > 2σ(*F*^2^)] = 0.022	H atoms treated by a mixture of independent and constrained refinement
*wR*(*F*^2^) = 0.058	*w* = 1/[σ^2^(*F*_o_^2^) + (0.0329*P*)^2^ + 0.0147*P*] where *P* = (*F*_o_^2^ + 2*F*_c_^2^)/3
*S* = 1.07	(Δ/σ)_max_ = 0.002
3449 reflections	Δρ_max_ = 0.25 e Å^−^^3^
100 parameters	Δρ_min_ = −0.17 e Å^−^^3^
1 restraint	Absolute structure: Flack (1983), 1600 Friedel pairs
Primary atom site location: structure-invariant direct methods	Flack parameter: 0.00 (3)

### Special details

**Table:**

Geometry. All e.s.d.'s (except the e.s.d. in the dihedral angle between two l.s. planes) are estimated using the full covariance matrix. The cell e.s.d.'s are taken into account individually in the estimation of e.s.d.'s in distances, angles and torsion angles; correlations between e.s.d.'s in cell parameters are only used when they are defined by crystal symmetry. An approximate (isotropic) treatment of cell e.s.d.'s is used for estimating e.s.d.'s involving l.s. planes.
Refinement. Refinement of *F*^2^ against ALL reflections. The weighted *R*-factor *wR* and goodness of fit *S* are based on *F*^2^, conventional *R*-factors *R* are based on *F*, with *F* set to zero for negative *F*^2^. The threshold expression of *F*^2^ > σ(*F*^2^) is used only for calculating *R*-factors(gt) *etc*. and is not relevant to the choice of reflections for refinement. *R*-factors based on *F*^2^ are statistically about twice as large as those based on *F*, and *R*- factors based on ALL data will be even larger.

### Fractional atomic coordinates and isotropic or equivalent isotropic displacement parameters (Å^2^)

**Table:**

	*x*	*y*	*z*	*U*_iso_*/*U*_eq_	
O1	0.19488 (14)	0.39944 (12)	0.93010 (6)	0.03194 (14)	
O2	0.32242 (18)	0.69384 (13)	0.84254 (7)	0.04120 (18)	
O3	0.57878 (12)	0.22667 (10)	0.72179 (7)	0.02923 (13)	
H3	0.644 (3)	0.119 (3)	0.6913 (14)	0.045 (4)*	
N1	0.25944 (14)	0.54258 (11)	0.63222 (7)	0.02279 (12)	
H1A	0.411 (3)	0.597 (3)	0.6391 (12)	0.037 (4)*	
H1B	0.254 (3)	0.473 (3)	0.5725 (15)	0.045 (4)*	
H1C	0.149 (2)	0.643 (2)	0.6261 (11)	0.029 (3)*	
C1	0.24364 (16)	0.51767 (14)	0.83958 (7)	0.02379 (14)	
C2	0.18095 (14)	0.40362 (12)	0.72813 (7)	0.02175 (13)	
H2	−0.0084	0.3827	0.7239	0.026*	
C3	0.31005 (16)	0.19224 (13)	0.71800 (8)	0.02470 (14)	
H3A	0.2571	0.1003	0.7821	0.030*	
H3B	0.2616	0.1242	0.6447	0.030*	
C4	0.2501 (2)	0.49492 (19)	1.04131 (9)	0.0378 (2)	
H4A	0.4322	0.5307	1.0456	0.057*	
H4B	0.2079	0.3961	1.1028	0.057*	
H4C	0.1473	0.6220	1.0502	0.057*	
Cl1	0.77391 (3)	0.81723 (3)	0.608559 (15)	0.02488 (4)	

### Atomic displacement parameters (Å^2^)

**Table:**

	*U*^11^	*U*^22^	*U*^33^	*U*^12^	*U*^13^	*U*^23^
O1	0.0416 (3)	0.0290 (3)	0.0252 (3)	−0.0042 (3)	0.0023 (2)	0.0007 (2)
O2	0.0668 (5)	0.0250 (3)	0.0317 (3)	−0.0129 (4)	−0.0020 (3)	−0.0044 (3)
O3	0.0229 (3)	0.0219 (3)	0.0429 (4)	0.0033 (2)	−0.0034 (2)	−0.0061 (2)
N1	0.0220 (3)	0.0201 (3)	0.0263 (3)	0.0021 (2)	−0.0026 (2)	−0.0013 (2)
C1	0.0236 (3)	0.0218 (3)	0.0260 (3)	0.0014 (3)	0.0001 (3)	−0.0023 (3)
C2	0.0187 (3)	0.0196 (3)	0.0270 (3)	−0.0016 (3)	−0.0014 (2)	−0.0012 (3)
C3	0.0250 (3)	0.0168 (3)	0.0323 (4)	−0.0016 (3)	−0.0027 (3)	−0.0025 (3)
C4	0.0485 (6)	0.0403 (5)	0.0248 (4)	0.0096 (4)	0.0003 (4)	−0.0030 (4)
Cl1	0.02321 (7)	0.02180 (7)	0.02965 (8)	0.00378 (7)	0.00166 (5)	0.00182 (8)

### Geometric parameters (Å, °)

**Table:**

O1—C1	1.3221 (11)	C1—C2	1.5235 (12)
O1—C4	1.4598 (13)	C2—C3	1.5153 (12)
O2—C1	1.1998 (11)	C2—H2	1.0000
O3—C3	1.4223 (10)	C3—H3A	0.9900
O3—H3	0.847 (18)	C3—H3B	0.9900
N1—C2	1.4852 (11)	C4—H4A	0.9800
N1—H1A	0.867 (16)	C4—H4B	0.9800
N1—H1B	0.825 (18)	C4—H4C	0.9800
N1—H1C	0.865 (14)		
			
C1—O1—C4	115.45 (8)	C3—C2—H2	108.5
C3—O3—H3	104.9 (11)	C1—C2—H2	108.5
C2—N1—H1A	115.1 (10)	O3—C3—C2	107.42 (6)
C2—N1—H1B	107.6 (12)	O3—C3—H3A	110.2
H1A—N1—H1B	108.9 (14)	C2—C3—H3A	110.2
C2—N1—H1C	108.6 (9)	O3—C3—H3B	110.2
H1A—N1—H1C	108.6 (14)	C2—C3—H3B	110.2
H1B—N1—H1C	107.7 (14)	H3A—C3—H3B	108.5
O2—C1—O1	125.48 (9)	O1—C4—H4A	109.5
O2—C1—C2	123.17 (8)	O1—C4—H4B	109.5
O1—C1—C2	111.33 (7)	H4A—C4—H4B	109.5
N1—C2—C3	110.58 (7)	O1—C4—H4C	109.5
N1—C2—C1	107.14 (6)	H4A—C4—H4C	109.5
C3—C2—C1	113.45 (7)	H4B—C4—H4C	109.5
N1—C2—H2	108.5		
			
C4—O1—C1—O2	−1.64 (14)	O2—C1—C2—C3	127.65 (10)
C4—O1—C1—C2	179.72 (7)	O1—C1—C2—C3	−53.67 (9)
O2—C1—C2—N1	5.33 (11)	N1—C2—C3—O3	59.87 (9)
O1—C1—C2—N1	−175.99 (7)	C1—C2—C3—O3	−60.53 (9)

### Hydrogen-bond geometry (Å, °)

**Table:**

*D*—H···*A*	*D*—H	H···*A*	*D*···*A*	*D*—H···*A*
N1—H1A···Cl1	0.867 (16)	2.390 (16)	3.2237 (8)	161.2 (14)
N1—H1B···Cl1^i^	0.825 (18)	2.336 (18)	3.1563 (8)	172.7 (16)
N1—H1C···Cl1^ii^	0.865 (14)	2.264 (14)	3.0979 (7)	161.9 (12)
O3—H3···Cl1^iii^	0.847 (18)	2.261 (18)	3.1041 (7)	173.9 (15)

Symmetry codes: (i) −*x*+1, *y*−1/2, −*z*+1; (ii) *x*−1, *y*, *z*; (iii) *x*, *y*−1, *z*.
